# Moderate Ischemic Mitral Regurgitation with Ejection Fraction <40% Undergoing Concomitant Mitral Valve Repair during Revascularization: A Single-Center Observational Study

**DOI:** 10.31083/j.rcm2411328

**Published:** 2023-11-24

**Authors:** Ye Yang, Fangyu Liu, Yulin Wang, Limin Xia, Chunsheng Wang, Qiang Ji

**Affiliations:** ^1^Department of Cardiovascular Surgery, Zhongshan Hospital Fudan University, 200032 Shanghai, China; ^2^Department of Cardiovascular Surgery, Xiamen Branch of Zhongshan Hospital Fudan University, 361015 Xiamen, Fujian, China; ^3^Shanghai Municipal Institute for Cardiovascular Diseases, 200032 Shanghai, China

**Keywords:** moderate ischemic mitral regurgitation, depressed left ventricular function, mitral valve repair, surgical revascularization

## Abstract

**Background:**

Numerous studies have examined the therapeutic effects of mitral valve repair during revascularization on moderate ischemic mitral regurgitation (IMR), as well as the incremental benefit of subvalvular repair alongside an annuloplasty ring. However, the impact of depressed left ventricular (LV) function on the surgical outcome of patients with moderate IMR has been rarely investigated. The aims of this single-center, retrospective, observational study were firstly to evaluate short- and medium-term outcomes in this patient group after undergoing mitral valve repair during revascularization, and secondly to assess the impact of depressed LV function on surgical outcomes.

**Methods:**

A total of 272 eligible patients who had moderate IMR and underwent concomitant mitral valve repair and revascularization from January 2010 to December 2017 were included in the study. These patients were categorized into different groups based on their ejection fraction (EF) levels: an EF <40% group (n = 90) and an EF ≥40% group (n = 182). The median time course of follow-up was 42 months and the shortest follow-up time was 30 months. This study compared in-hospital outcomes (major postoperative morbidity and surgical mortality) as well as midterm outcomes (moderate or more mitral regurgitation, all-cause mortality, and reoperation) of the two groups before and after propensity score (PS) matching (1:1).

**Results:**

No significant difference was observed in surgical mortality between groups (8.9% *vs.* 3.3%, *p* = 0.076). More patients in the EF <40% group developed low cardiac output (8.9% *vs.* 2.7%, *p* = 0.034) and prolonged ventilation (13.3% *vs. *5.5%, *p* = 0.026) compared to the EF ≥40% group. Propensity score (PS) matching successfully established 82 patient pairs in a 1:1 ratio. No significance was discovered between the matched cohorts in terms of major postoperative morbidity and surgical mortality, except for prolonged ventilation. Conditional mixed-effects logistic regression analysis revealed that EF <40% had an independent impact on prolonged ventilation (odds ratio (OR) = 2.814, 95% CI 1.321–6.151, *p* = 0.031), but was not an independent risk factor for surgical mortality (OR = 2.967, 95% CI 0.712–7.245, *p* = 0.138) or other major postoperative morbidity. Furthermore, the two groups showed similar cumulative survival before (log-rank *p* = 0.278) and after (stratified log-rank *p* = 0.832) PS matching. *Cox* regression analysis suggested that EF <40% was not related to mortality compared with EF ≥40% (PS-adjusted hazard ratio (HR) = 1.151, 95% CI 0.763–1.952, *p* = 0.281).

**Conclusions:**

Patients with moderate IMR and EF <40% shared similar midterm outcomes and surgical mortality to patients with moderate IMR and EF ≥40%, but received prolonged ventilation more often. Depressed LV function may be not associated with surgical or midterm mortality.

## 1. Introduction

Ischemic mitral regurgitation (IMR) affects over two million individuals in the United States and is the most frequent etiology of functional mitral regurgitation (MR) [1]. IMR is provoked by acute or chronic coronary artery disease. The tethering mitral leaflets appear with unsatisfactory coaptation, predominantly as a result of disadvantageous left ventricular (LV) remodeling with annular dilatation [2]. The risks of death and heart failure increase with the development of IMR and increase further with the severity of regurgitation [3]. Because the shaping of adverse LV remodeling may vary, IMR also shows diversity according to distinct LV injuries. Even when small ischemic or infarcted areas appear, especially in the posterolateral region, obvious IMR occurs despite the ventricle showing good performance overall [4]. Dynamic, paroxysmal MR patterns often exist in such patients, who are thought to benefit from surgery. Some patients also have severely dilated ventricles, usually accompanied by low ejection fraction (EF). The outcomes for these patients are often disappointing and unpredictable [5]. EF is an evaluation index for LV systolic performance and has decision-making value in the treatment of IMR [6, 7]. Ellis *et al*. [8] conducted an observational study of 3-year survival following percutaneous coronary intervention in IMR patients. These authors found that depressed LV function (EF <40%) may be related to increased 3-year mortality.

According to the guidelines from the American Association for Thoracic Surgery, mitral valve repair using an undersized ring annuloplasty is recommended as “may be considered” for moderate IMR during surgical revascularization [9]. An increasing number of studies have reported that coronary artery bypass grafting (CABG) plus simultaneous mitral valve repair could be an effective surgical plan for moderate IMR, although recurrent MR may occur. Surgery eliminates MR immediately following the operation, reverses LV remodeling, ameliorates LV performance, and allows a more reliable repair of moderate IMR [10, 11, 12]. The results of our previous study showed that concomitant mitral valve repair may improve New York Heart Association (NYHA) functional class and reduce residual MR, with no increase in surgical mortality, morbidity, or follow-up deaths [13].

Previous studies assessed mainly the therapeutic effects of revascularization along with mitral valve repair in moderate IMR patients, and the incremental benefits of subvalvular repair plus an annuloplasty ring. However, the impact of depressed LV function on surgical outcomes of patients with moderate IMR has not been determined. Based on our experience, we hypothesize that concomitant mitral valve repair during revascularization is safe, feasible, and effective in patients with moderate IMR and EF <40%. The aims of this present study included the evaluation of short- and medium-term outcomes for patients with moderate IMR and EF <40% who received mitral valve repair during CABG, and the assessment of the impact of depressed LV function on surgical outcomes.

## 2. Materials and Methods

### 2.1 Patient Characteristics

Consecutive patients with moderate IMR and scheduled for mitral valve repair and simultaneous CABG between January 2010 to December 2017 were identified from medical records. The inclusion criteria were: (1) previous myocardial infarction (MI) indicated by regional wall motion abnormalities revealed by echocardiography, or as detected by electrocardiogram; (2) sinus rhythm; and (3) no structural mitral valve abnormalities. The exclusion criteria were: (1) echocardiographic evidence and/or clinical manifestations of other structural heart diseases; (2) organic mitral apparatus abnormalities; (3) unstable global clinical status; (4) atrial fibrillation that was not appropriate for this study because it was reported to cause atrioventricular valve regurgitation [14]; (5) concomitant tricuspid annuloplasty; and (6) emergency surgery.

Within 3 days before surgery, routine transthoracic echocardiography was performed to assess the severity and mechanism of MR. The IMR level was classified as mild (narrow central jet area less than 20% of left atrium (LA) and vena contracta less than 3.0 mm under Doppler), moderate (regurgitant volume less than 30 mL, effective regurgitant orifice area (EROA) less than 20 ${\mathrm{mm}}^{2}$, and regurgitant fraction less than 50%), or severe (regurgitant volume over 30 mL, EROA over 20 ${\mathrm{mm}}^{2}$, and regurgitant fraction over 50%). MR suggested by echocardiography was evaluated by two independent professional readers, with a third reader used when discrepancies arose. Examinations were performed according to current guidelines [15].

All procedures began with a midline sternotomy. The detailed protocol for on-pump CABG was described in a previous study [16]. After grafting, the quality of anastomosis was evaluated during the operation using a transit-time flow probe. Technical details regarding our institutional approach to mitral valve repair were described previously [17]. After weaning off the bypass, intraoperative transesophageal echocardiography was performed immediately to determine the quality of mitral valve repair. If moderate or more residual MR was observed, a repeat procedure was executed immediately.

### 2.2 Study Protocol

This single-center, retrospective, observational study received approval from the medical ethics committee of Zhongshan Hospital, Fudan University (No. B2022-024R), and followed the principles of the Declaration of Helsinki. Similar to a previous report [8] in which an EF of 40% was used as the cut-off point to discriminate depressed LV function, EF <40% was used in the present study to define depressed LV function. All patients included in this study were assigned to either the EF <40% group or the EF ≥40% group. Baseline characteristics and surgical data were extracted for all patients, and in-hospital and follow-up outcomes were compared between groups. An electronic in-hospital database was used to access baseline characteristics, as well as in-hospital outcomes. A standard case report form was used to record the data. Enrolled patients were routinely followed up postoperatively at three and six months, and afterward at 6-month intervals. Telephone interviews and clinical visits were used to obtain follow-up data. A clinical visit was scheduled if recrudescent symptoms of coronary artery disease or questionable symptoms of MR appeared during the follow-up.

In-hospital outcomes consisted of surgical mortality and major postoperative morbidity. Death during the same hospitalization or within 30 days after surgery would be recognized as surgical mortality [18]. Major postoperative morbidities were CABG-associated MI, prolonged ventilation, low cardiac output, new-onset stroke, acute kidney injury requiring hemodialysis, redo for bleeding, and deep sternal wound infection (DSWI). CABG-associated MI was diagnosed as elevation of cardiac troponin T (cTnT) values to >10 times the 99th percentile of the upper reference range using one or more of the following methods: (1) new onset pathological Q wave or left bundle branch block recorded with electrocardiography; (2) new occlusion of graft or native coronary artery documented by angiography; (3) new viable myocardium loss or abnormal regional wall motion manifested by imaging [19]. Low cardiac output was recorded when an intra-aortic balloon pump (IABP) and/or positive inotropic agent support became necessary for difficulty in weaning off the cardiopulmonary bypass, or when longer than 30 mins was required to maintain the cardiac index >2.2 L/min/$m^{2}$ with systolic blood pressure >90 mmHg after the patient returned to intensive care unit (ICU) [20]. Postoperative prolonged ventilation was identified as when the mechanical ventilation exceeded 48 hours, or when re-intubation was required after surgery. New-onset stroke was considered to be a new onset of global or focal brain dysfunction occurring over 24 hours, or permanent neurological damage persisting until either discharge or death [21]. The definition of DSWI was the same as in our previous study [22]. The duration of the postoperative hospital stay and of the ICU stay were counted.

Follow-up outcomes included all-cause death, reoperation (including repeat mitral valve procedure and repeat revascularization), moderate or severe MR, and NYHA functional class. All-cause mortality is the most unbiased and robust index and was therefore selected rather than cardiac mortality. This helped to avoid misinterpretation of the cause of death due to unreliable medical records. The minimum follow-up time in this study was 30 months. Follow-up information obtained at 30 months after surgery was used for the analysis of NYHA classification and residual MR.

### 2.3 Statistical Analysis

The normal distribution of variables was determined using the Shapiro-Wilks test. An independent-samples *t*-test was used while comparing normally distributed continuous variables between groups, which were exhibited as the mean ± standard deviation (SD). The Wilcoxon rank sum test was performed on non-normally distributed continuous variables, which were exhibited as the median and interquartile range (IQR). Categorical variables were exhibited as frequencies and percentages, which were compared using the Chi-square test between groups, or using the Fisher’s exact test when the expected frequency was <5.

The propensity score (PS) was generated for each patient using a multivariable logistic regression model to control potential confounders in the dataset. The PS was performed according to 17 independent variables, with LV function-based grouping (EF <40% *vs*. EF ≥40%) used as a binary dependent variable. Demographics, complications, cardiac status (except for EF and EuroSCORE), and EROA were included in the 17 variables. The model was verified with the Hosmer-Lemeshow goodness-of-fit method. The greedy-matching algorithm was used with a caliper width of 0.2 of the SD of the logit of the PS, thus implementing a 1:1 nearest-neighbor. Other details of the PS matching could refer to our former study [16]. After matching, paired *t*-test was used for normally distributed continuous variates. The Wilcoxon signed-rank test was used for non-normally distributed continuous variates. McNemar’s test was used for categorical variates. Conditional mixed-effects logistic regression analysis was applied to evaluate the effect of grouping as independent risk factors. The Kaplan-Meier method was applied to estimate the overall survival and survival free from reoperation with the stratified log-rank test used to compare survival curves in the PS-matched population. Between the two matched groups, the *Cox* regression model was utilized to estimate the PS-adjusted hazard ratio (HR) and 95% confidence interval (CI) of midterm mortality. Statistical analysis was performed using SPSS version 22.0 (SPSS Inc., Chicago, IL, USA), with a two-sided *p*-value < 0.05 considered to represent statistical significance.

## 3. Results

### 3.1 Study Population

**Fig. 1.**
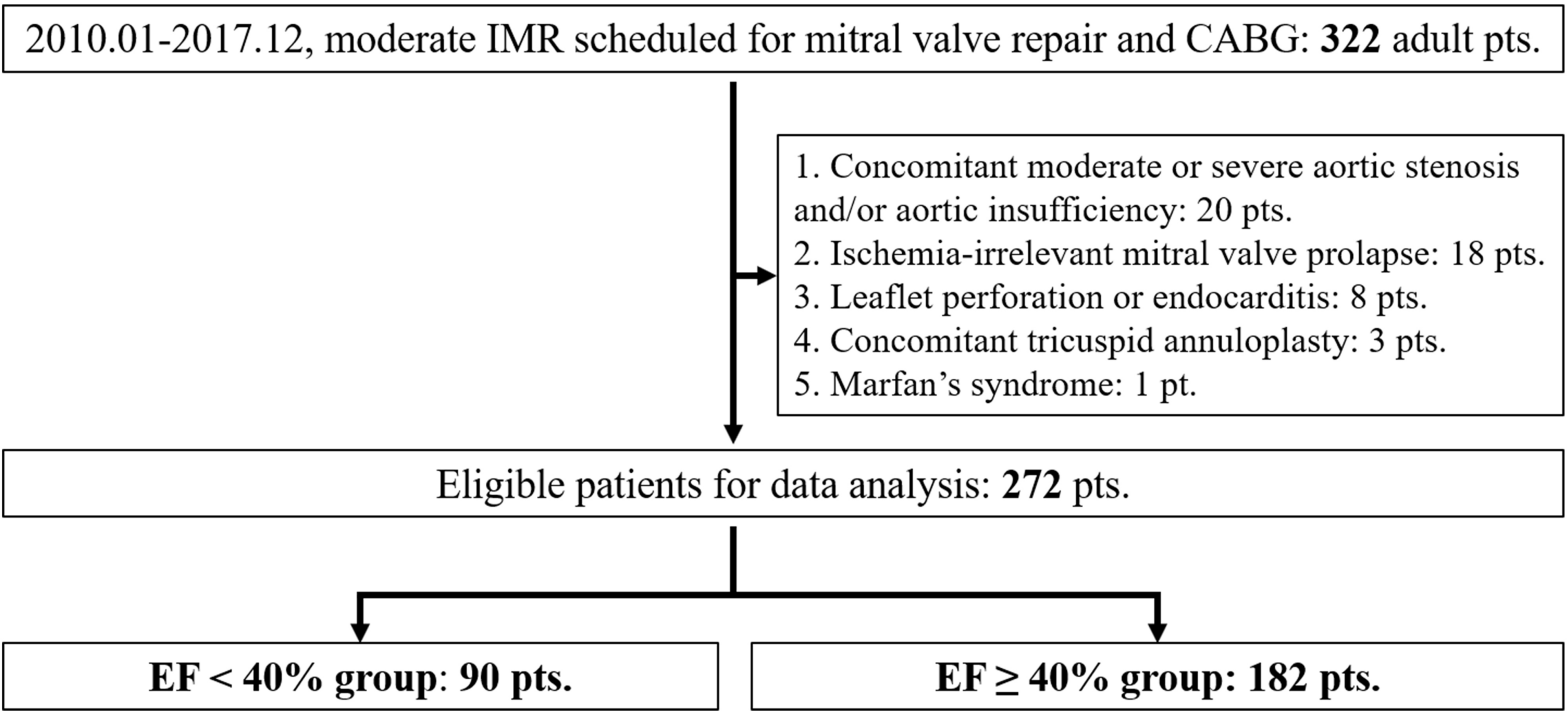
**Flow chart for the patient enrollment**. IMR, ischemic mitral regurgitation; CABG, coronary artery bypass grafting; pts, patients; EF, ejection fraction.

A total of 5336 consecutive patients in our department received surgical revascularization with or without other concomitant cardiac surgery between January 2010 to December 2017. Amongst these, 322 patients were eligible according to the inclusion criteria. During patient enrollment, as shown in Fig. 1, 50 patients were ruled out, leaving 272 patients for data analysis. Of these, 90 patients were enrolled in the EF <40% group, and 182 patients in the EF ≥40% group.

Baseline characteristics of the patients are shown in Table 1. Patients in the EF <40% group had a larger LV endo-diastolic diameter (64.5 ± 7.8 mm *vs*. 59.2 ± 7.1 mm, *p* < 0.001), a higher proportion of patients in NYHA class III–IV (*p* = 0.023), and a higher additive EuroSCORE (*p* = 0.012) compared with patients in the EF ≥40% group. Baseline characteristics were otherwise similar between groups.

**Table 1.** **Baseline characteristics**.

Variables		Unmatched population			Matched population		
		EF <40% (n = 90)	EF ≥40% (n = 182)	*p*	EF <40% (n = 82)	EF ≥40% (n = 82)	*p*
Demographics							
	Age (years)	64.1 ± 7.8	65.3 ± 8.3	0.254	64.4 ± 7.8	65.0 ± 8.1	0.630
	Gender (female)	15, 16.7%	35, 19.2%	0.607	14, 17.1%	15, 18.3%	0.838
	Obesity	14, 15.6%	26, 14.3%	0.781	12, 14.6%	13, 15.9%	0.828
	Smoking history	42, 46.7%	78, 42.9%	0.552	38, 46.3%	36, 43.9%	0.754
Concomitant diseases							
	Hypertension	47, 52.2%	94, 51.6%	0.929	43, 52.4%	42, 51.2%	0.876
	Diabetes	40, 44.4%	75, 41.2%	0.611	37, 45.1%	35, 42.7%	0.753
	Hyperlipidemia	20, 22.2%	41, 22.5%	0.955	18, 22.0%	17, 20.7%	0.849
	CKD	8, 8.9%	17, 9.3%	0.903	7, 8.5%	9, 11.0%	0.599
	Prior CVA	7, 7.8%	15, 8.2%	0.895	6, 7.3%	7, 8.5%	0.773
	COPD	7, 7.8%	21, 11.5%	0.337	6, 7.3%	9, 11.0%	0.416
Preoperative cardiac status							
	Recent MI	38, 43.3%	75, 41.2%	0.873	36, 43.9%	34, 41.5%	0.752
	Previous PCI	15, 16.7%	29, 15.9%	0.877	13, 15.9%	12, 14.6%	0.828
	NYHA III–IV	32, 35.6%	41, 22.5%	0.023	30, 36.6%	22, 26.8%	0.179
	EF	0.38 ± 0.04	0.49 ± 0.09	<0.001	0.38 ± 0.03	0.48 ± 0.05	<0.001
	LVEDD (mm)	64.5 ± 7.8	59.2 ± 7.1	<0.001	64.1 ± 7.2	62.0 ± 6.7	0.055
	EROA (${\mathrm{mm}}^{2}$)	16 (12, 18)	16 (12, 17)	0.452	16 (12, 18)	16 (12, 17)	0.408
Extent of CAD							
	2-vessel	12, 13.3%	28, 15.4%	0.653	11, 13.4%	12, 14.6%	0.822
	3-vessel	78, 86.7%	154, 84.6%		71, 86.6%	70, 85.4%	
	LM	26, 28.9%	54, 29.7%	0.894	24, 29.3%	25, 30.5%	0.865
EuroSCORE		7 (5, 8)	7 (5, 7)	0.012	7 (5, 8)	7 (5, 8)	0.201

CKD, chronic kidney disease; CVA, cerebro-vascular accident; COPD, chronic obstructive pulmonary disease; MI, myocardial infarction; PCI, percutaneous coronary intervention; NYHA, New York Heart Association; EF, ejection fraction; LVEDD, left ventricular endo-diastolic diameter; EROA, effective regurgitant orifice area; CAD, coronary artery disease; LM, left main trunk disease.

Surgical data are shown in Table 2. No significant difference was discovered between groups in terms of cardiopulmonary bypass time or aortic cross-clamping time. The number of grafts was also similar between the two groups (*p* = 0.653). Annuloplasty was performed on 232 patients using a downsized complete rigid ring for 84.4% of the EF <40% group and 85.7% of the EF ≥40% group (*p* = 0.781). No significant difference was apparent for the type of repair techniques used in the two groups (*p* = 0.667). Transesophageal echocardiography examination revealed that moderate or severe MR was not found in either group immediately after weaning off the bypass.

**Table 2.** **Surgical data**.

Variables		Unmatched population			Matched population		
		EF <40% (n = 90)	EF ≥40% (n = 182)	*p*	EF <40% (n = 82)	EF ≥40% (n = 82)	*p*
CPB time (min)		97.8 ± 21.4	91.7 ± 20.1	0.071	97.5 ± 21.1	93.4 ± 19.1	0.194
ACC time (min)		77.5 ± 12.1	76.8 ± 12.9	0.721	77.3 ± 12.0	76.5 ± 11.8	0.663
Number of grafts		3 (3, 3)	3 (3, 3)	0.653	3 (3, 3)	3 (3, 3)	0.712
Use of left IMA		89, 98.9%	180, 98.9%	0.993	82, 100%	81, 98.8%	0.316
Use of vein graft		88, 97.8%	177, 97.3%	0.797	81, 98.8%	81, 98.8%	>0.999
Use of RA		5, 5.6%	6, 3.3%	0.514	5, 6.1%	3, 3.7%	0.720
Type of ring							
	Band	14, 15.6%	26, 14.3%	0.781	11, 13.4%	12, 14.6%	0.822
	Complete-ring	76, 84.4%	156, 85.7%		71, 86.6%	70, 85.4%	
Size of complete-ring		28 (28, 30)	28 (26, 30)	0.572	28 (28, 30)	28 (28, 30)	0.718
Repair techniques							
	Annuloplasty alone	64, 71.1%	142, 78.1%	0.667	61, 74.4%	63, 76.8%	0.972
	Plus sub-valvular	11, 12.3%	17, 9.3%		9, 11.0%	9, 11.0%	
	Plus leaflet	13, 14.4%	20, 11.0%		11, 13.4%	9, 11.0%	
	All	2, 2.2%	3, 1.6%		1, 1.2%	1, 1.2%	
TEE data							
	No or trace MR	81, 90.0%	162, 89.0%	0.804	76, 92.7%	75, 91.5%	0.773
	Mild MR	9, 10.0%	20, 11.0%		6, 7.3%	7, 8.5%	

CPB, cardiopulmonary bypass; ACC, aortic cross-clamping; IMA, internal mammary artery; RA, radial artery; TEE, transesophageal echocardiography; MR, mitral regurgitation; EF, ejection fraction.

### 3.2 Propensity Score Matching Cohorts

**Fig. 2.**
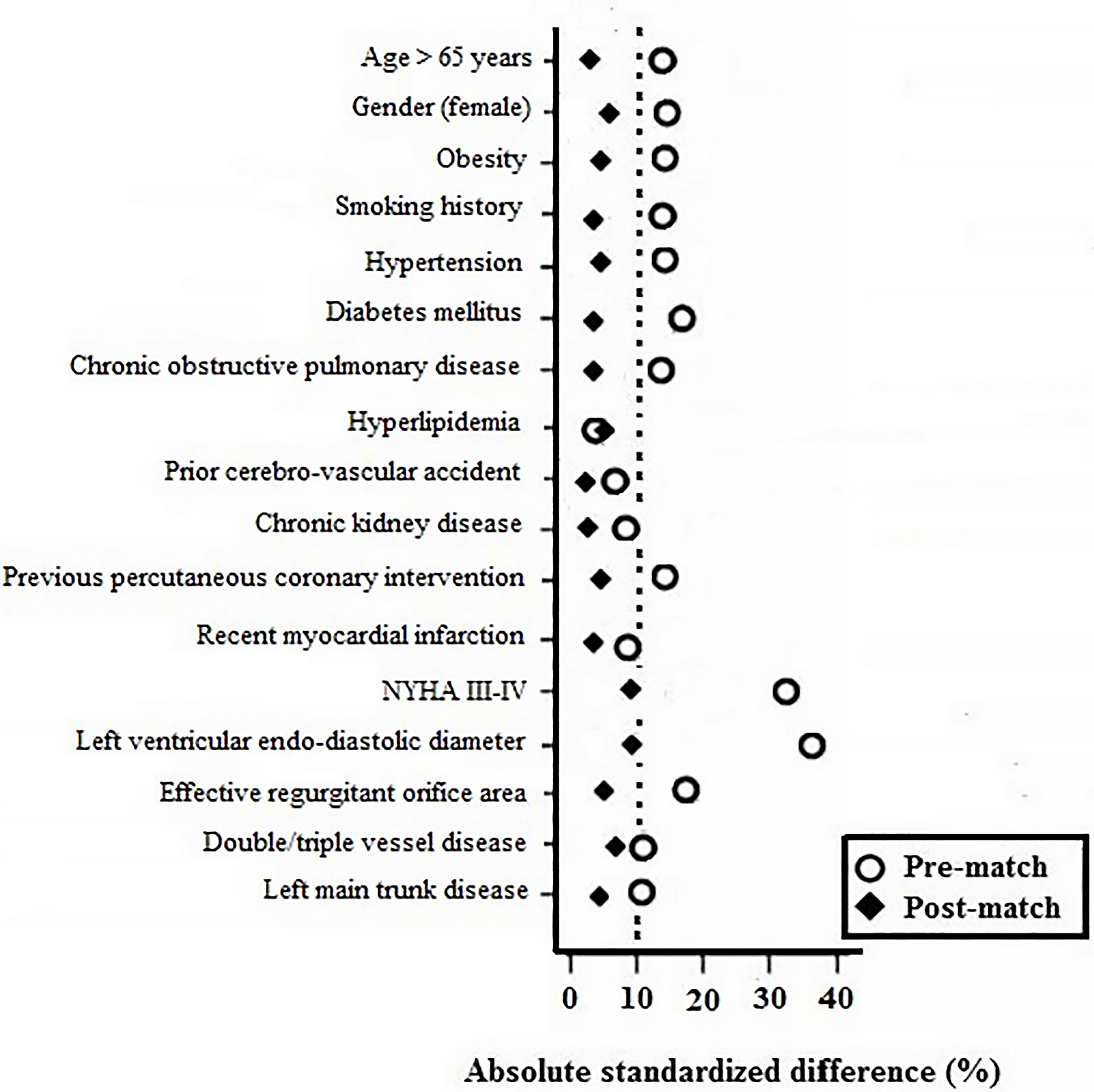
**Pre-match and post-match absolute standardized differences for baseline characteristics**. NYHA, New York heart assessment functional classification.

To compare baseline characteristics between the two groups, we performed bivariate analyses. The propensity score was calculated based on 17 predefined variables. The model’s Hosmer-Lemeshow goodness of fit was 4.65 (*p* = 0.793). Furthermore, good discrimination power was achieved with an area under the curve of 0.78 (95% CI, 0.65–0.84, *p* = 0.019) of the receiver operating curve. Ultimately, 82 patient pairs were matched at a 1:1 ratio. After matching, Fig. 2 shows that the absolute standardized differences were all <10%, which indicates adequate balance. Except for EF, the matched cohorts were comparable for baseline characteristics (Table 1). In addition, no significant difference was found with regard to surgical characteristics between the two matched groups.

### 3.3 In-Hospital Outcomes

The in-hospital outcomes are shown in Table 3. Patients in the EF <40% group had slightly higher surgical mortality, but this reached no statistical significance (*p* = 0.076). More patients in the EF <40% group developed low cardiac output (8.9% *vs*. 2.7%, *p* = 0.034) and received intra-aortic balloon pump support (10.0% *vs*. 3.8%, *p *= 0.042) compared to patients in the EF ≥40% group. More patients in the EF <40% group also developed prolonged ventilation (13.3% *vs*. 5.5%, *p* = 0.026). Major postoperative morbidity was otherwise similar between the two groups, including CABG-associated MI, redo for bleeding, new-onset stroke, DSWI, and acute kidney injury requiring hemodialysis. The matched groups of patients showed similar major postoperative morbidity, with the exception of prolonged ventilation. Patients in the EF <40% group had longer ICU stays and longer postoperative hospital stays compared with those in the EF ≥40% group, both before and after PS matching.

**Table 3.** **Clinical Outcomes**.

Variables		Unmatched population			Matched population		
		EF <40%	EF ≥40%	*p*	EF <40%	EF ≥40%	*p*
In-hospital							
	Number of patients	90	182		82	82	
	Surgical mortality	8, 8.9%	6, 3.3%	0.076	7, 8.5%	2, 2.4%	0.167
	CABG-associated MI	3, 3.3%	5, 2.7%	0.722	2, 2.4%	1, 1.2%	>0.999
	Low cardiac output	8, 8.9%	5, 2.7%	0.034	7, 8.5%	2, 2.4%	0.167
	IABP support	9, 10.0%	7, 3.8%	0.042	8, 9.8%	3, 3.7%	0.119
	Redo for bleeding	3, 3.3%	6, 3.3%	0.999	2, 2.4%	2, 2.4%	>0.999
	New-onset stroke	4, 4.4%	5, 2.7%	0.484	3, 3.7%	2, 2.4%	>0.999
	Prolonged ventilation	12, 13.3%	10, 5.5%	0.026	11, 13.4%	3, 3.7%	0.025
	DSWI	2, 2.2%	3, 1.6%	0.667	1, 1.2%	1, 1.2%	>0.999
	AKI requiring hemodialysis	6, 6.7%	5, 2.7%	0.187	5, 6.1%	2, 2.4%	0.443
	ICU stay (d)	4 (2, 5)	2 (1, 3)	<0.001	3 (2, 4)	2 (2, 3)	0.012
	Postoperative hospital stay (d)	8 (7, 10)	7 (6, 8)	<0.001	8 (7, 10)	7 (6, 9)	0.009
Follow-up							
	Number of patients	78	165		74	75	
	Follow-up time (months)	42 (36, 48)	42 (34, 50)	0.318	42 (37, 48)	43 (36, 50)	0.101
At 30-month							
	Moderate or more MR	19, 24.4%	35, 21.2%	0.582	18, 24.3%	16, 21.3%	0.664
	NYHA III–IV	11, 14.1%	19, 11.5%	0.567	9, 12.2%	8, 10.7%	0.774

EF, ejection fraction; CABG, coronary artery bypass grafting; MI, myocardial infarction; IABP, intra-aortic balloon pump; DSWI, deep sternal wound infection; AKI, acute kidney injury; ICU, intensive care unit; MR, mitral regurgitation; NYHA, New York Heart Association.

The effects of grouping (matched EF <40% group *vs*. matched EF ≥40% group) on major postoperative morbidity and surgical mortality are shown in Table 4. Conditional mixed-effects logistic regression analysis revealed that EF <40% had an independent impact on postoperative prolonged ventilation (OR = 2.814, 95% CI 1.321–6.151, *p* = 0.031). However, EF <40% was not an independent risk factor for surgical mortality (OR = 2.967, 95% CI 0.712–7.245, *p* = 0.138), nor was it an independent risk factor for other major postoperative morbidities.

**Table 4.** **Impacts of EF <40% on outcomes in the matched population**.

Outcomes	Univariate analysis		Multivariate analysis*	
	OR (95% CI)	*p*	OR (95% CI)	*p*
Surgical mortality	3.733 (0.752–9.542)	0.086	2.967 (0.712–7.245)	0.138
CABG-associated MI	2.025 (0.580–7.780)	0.560	/	
Low cardiac output	3.562 (0.724–8.939)	0.105	3.134 (0.658–8.623)	0.214
Redo for bleeding	1.001 (0.407–6.274)	0.987	/	
New-onset stroke	1.519 (0.547–6.338)	0.650	/	
Prolonged ventilation	3.538 (1.116–7.215)	0.027	2.814 (1.321–6.151)	0.031
IABP support	2.847 (0.728–7.141)	0.183	2.634 (0.564–6.571)	0.310
DSWI	1.001 (0.123–7.263)	0.991	/	
AKI requiring hemodialysis	2.529 (0.589–7.790)	0.196	2.428 (0.634–5.578)	0.283
Moderate or more MR at 30 months	1.196 (0.558–2.623)	0.700	/	
NYHA III-IV at 30 months	1.162 (0.469–3.189)	0.801	/	

*The conditional mixed-effects logistic regression model included five variates with *p* < 0.20 in the univariate analysis. CABG, coronary artery bypass grafting; MI, myocardial infarction; IABP, intra-aortic balloon pump; DSWI, deep sternal wound infection; AKI, acute kidney injury; MR, mitral regurgitation; NYHA, New York Heart Association; OR, odds ratio; CI, confidence interval; EF, ejection fraction.

### 3.4 Follow-up Outcomes

**Fig. 3.**
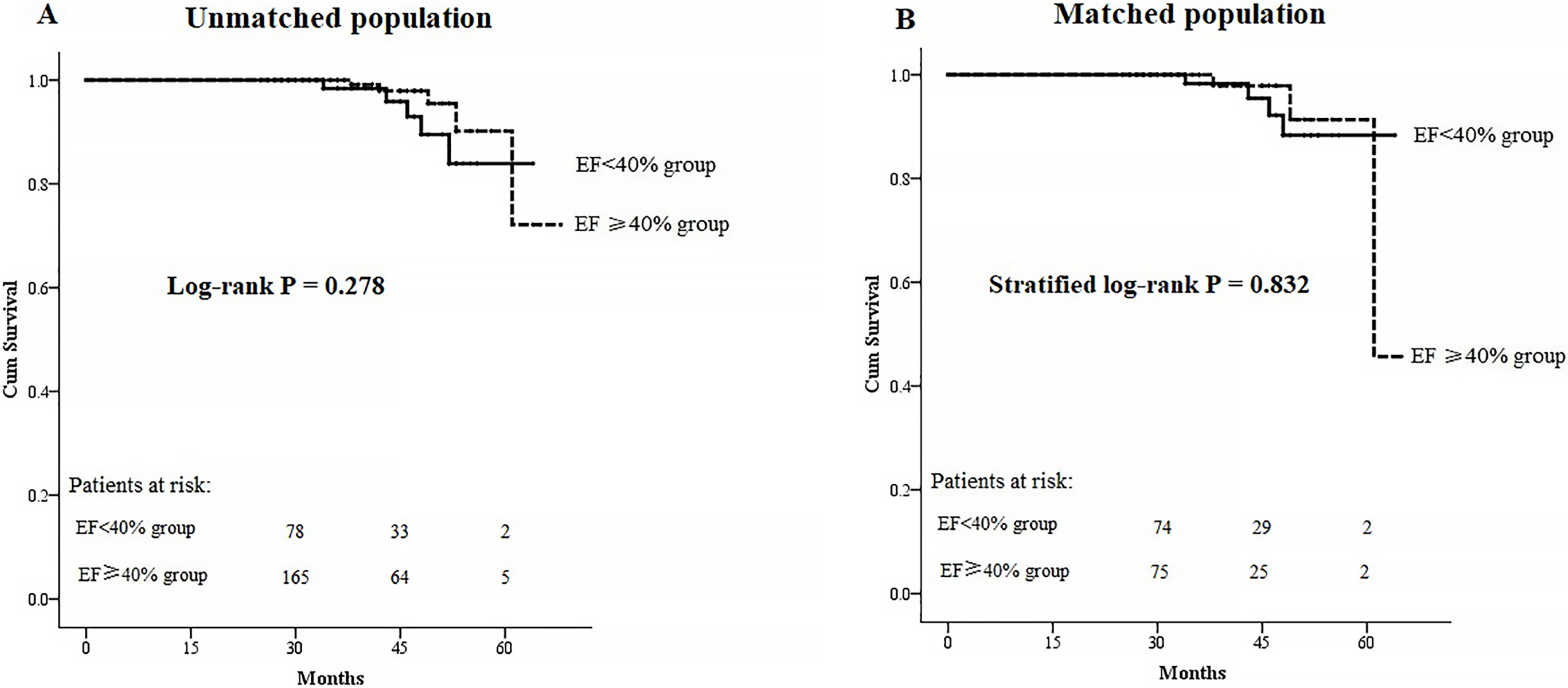
**Kaplan-Meier curves for overall survival**. (A) Kaplan-Meier curves in the unmatched cohorts. (B) Kaplan-Meier curves in the matched cohorts. EF, ejection fraction.

**Fig. 4.**
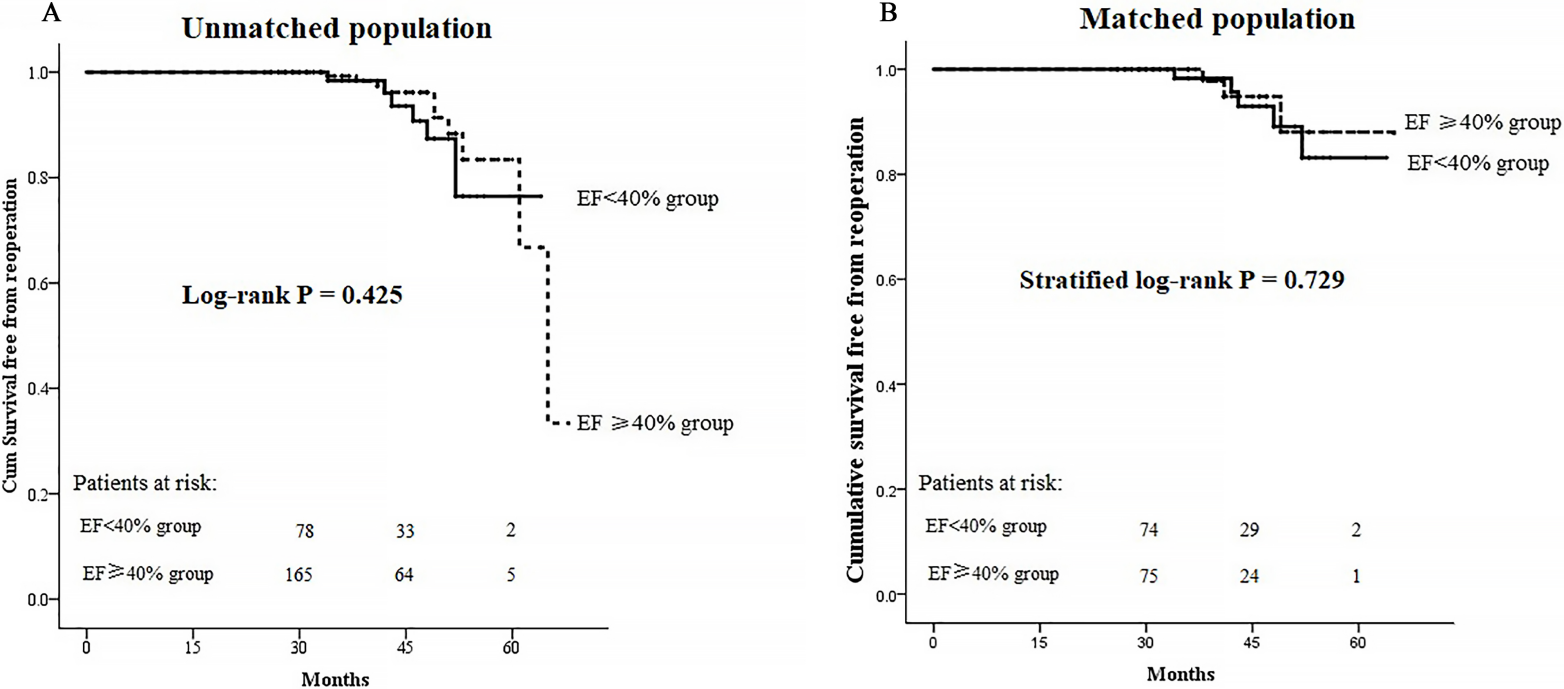
**Kaplan-Meier curves for survival free from reoperation**. (A) Kaplan-Meier curves in the unmatched cohorts. (B) Kaplan-Meier curves in the matched cohorts. EF, ejection fraction.

**Fig. 5.**
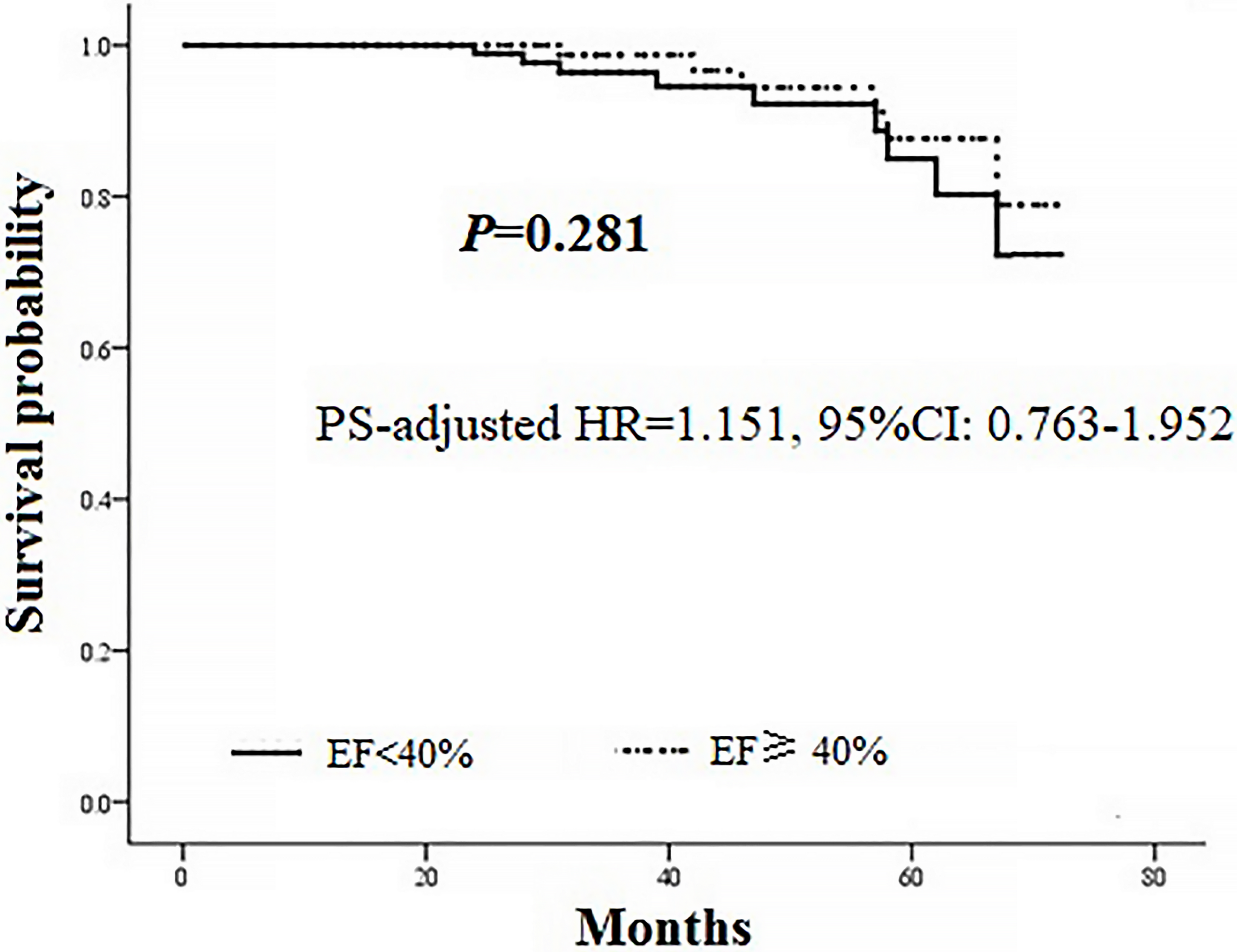
**PS-adjusted *Cox* regression analysis in the matched population**. PS, propensity score; HR, hazard ratio; CI, confidence interval; EF, ejection fraction.

Follow-up visits were completed with 243 patients in total. The median follow-up time was 42 months (IQR, 34–50), with the shortest follow-up time being 30 months. At the 30-month follow-up, the incidence of moderate or more MR and the proportion of NYHA class III and IV did not differ either before or after matching (Table 3). As shown in Fig. 3, similar cumulative survival was shown both before and after PS matching (log-rank *p* = 0.278, stratified log-rank *p* = 0.832, respectively). No significant difference in cumulative survival free from reoperation was found between the two groups, either before or after matching (log-rank *p* = 0.425, stratified log-rank *p* = 0.729, respectively) (Fig. 4). Finally, Cox regression analysis was utilized to estimate the follow-up death in the matched cohorts. As shown in Fig. 5, grouping based on EF (EF <40%* vs. *EF ≥40%) was not related to midterm mortality (PS-adjusted HR 1.151, 95% CI 0.763–1.952, *p* = 0.281).

## 4. Discussion

Valvular functional insufficiency in IMR is mainly attributed to disadvantageous LV remodeling and annular dilatation following myocardial injury, thereby resulting in poor coaptation of tethering mitral leaflets. Because the degree of LV remodeling can vary, IMR occurs within a broad range of LV injuries. An increasing number of studies have reported that mitral valve repair (ring annuloplasty, leaflet augmentation, subvalvular manipulation, or a combination of these) during surgical revascularization is adequate therapy for patients with moderate IMR [10, 12, 23, 24, 25, 26, 27]. However, few studies have evaluated the effect of depressed LV function on surgical outcomes in this patient group. We cannot be certain whether depressed LV function secondary to LV injury has negative impacts on the surgical outcome of this patient group. Several previous studies have hypothesized that EF may not reflect the true function of the left ventricle under several pathophysiological conditions, which could mask further weakened LV performance in patients with severe MR [28, 29, 30]. However, in the present study, we focused only on patients with moderate IMR, which is unlikely to have a significant effect on the EF. In patients with IMR, a lower EF could mostly be secondary to reduced LV contractility [31]. As per a previous report [8], in the current study we defined depressed LV function as EF <40%. Our goal was to evaluate the in-hospital and midterm outcomes of moderate IMR patients with EF <40% who received mitral valve repair during surgical revascularization, and secondly to evaluate the impacts of depressed LV function on surgical outcomes.

The key findings of our study were that, compared to moderate IMR patients with EF ≥40%, patients with EF <40% had similar midterm outcomes, a similar incidence of moderate or more MR, a similar proportion of NYHA class III-IV, and similar cumulative survival and cumulative survival free from reoperation. Furthermore, *Cox* regression analysis showed that EF grouping (EF <40% *vs*. EF ≥40%) was not associated with midterm mortality. The present results suggested that depressed LV function prior to surgery was not associated with any significant disadvantage in terms of midterm survival or NYHA functional status. Previously, Ellis *et al*. [8] found that depressed EF might be associated with increased 3-year mortality in IMR patients who received percutaneous coronary intervention. In contrast to the findings of our study, these authors speculated that depressed LV function could decrease the survival of patients with IMR who received percutaneous coronary intervention. This discrepancy may be due to differences in the study populations. The study by Ellis *et al*. [8] was on IMR patients who underwent percutaneous coronary intervention, whereas the present study was conducted on moderate IMR patients who received mitral valve repair during surgical revascularization. Using quantitative methods for functional MR grading, Rossi *et al*. [32] recently found that quantitatively defined functional MR was associated with the prognosis of patients with heart failure. This result concurred with the findings of the present study.

With regard to in-hospital outcomes, the current study found that patients with EF <40% had similar surgical mortality and other major postoperative morbidities to moderate IMR patients with EF ≥40%, but were more inclined to require prolonged ventilation after surgery. This result was validated using multivariable regression analysis and implied that depressed LVEF could contribute to poor postoperative respiratory function, consistent with the findings of earlier studies [33, 34]. Importantly, our study indicated that there was no association between depressed LV function prior to surgery and surgical mortality, consistent with results from other trials [35, 36, 37, 38]. Within patients with reduced LVEF and moderate to severe IMR, the additional mitral valve repair beyond CABG could also improve survival [39]. This could be due to the introduction over the past few years of advanced surgical techniques, perioperative management with new medicines, and assisted devices [40, 41, 42]. In addition, our study suggested that depressed LV function prior to surgery was not invariably linked to other major postoperative morbidities such as low cardiac output and acute kidney injury requiring hemodialysis. This implied that depressed LV function before surgery was not related to the deterioration of cardiac and renal function.

In general, EF <40% does not appear to be a contraindication for mitral valve repair during revascularization. The benefits of performing mitral interventions beyond CABG have been demonstrated in several studies. A randomized clinical trial of additional mitral valve repair for moderate IMR patients found greater improvements in oxygen consumption, MR severity, and LV remodeling [37]. In another trial, concomitant mitral valve repair resulted in better NYHA functional class, LV dimensions, LV function, and pulmonary artery pressure [38]. In the present study of patients with moderate IMR and depressed LV function, we observed favorable midterm outcomes and no increase in surgical mortality or major adverse cardiac events, although a higher incidence of prolonged ventilation after surgery was observed. These findings supported mitral valve repair during the revascularization of such patients.

Patients in the EF <40% group had larger LV diameters than patients in the EF ≥40% group, although this difference was reduced in the matched population. LV diameter was also an evaluation criterion for heart function. Enlarged LV suggested cardiac overload and can result from many types of heart disease. With lower EF, IMR patients may also experience more advanced LV diastolic dysfunction and heavier LV preload. Low EF and enlarged LV were both indicators of unfavorable clinical status. However, these patients could also benefit from surgery and still had satisfactory survival.

There were some limitations with the current study. First, the investigation was conducted in a single-center observational setting with a relatively small number of participants and relatively short follow-up, which could therefore affect the generalizability of the findings. Although no significant difference in surgical mortality was found between groups, the sample size may have limited the statistical power. Larger multicenter trials with longer follow-up times were required to further assess long-term outcomes in moderate IMR patients with depressed LV function who receive mitral valve repair during CABG, as well as the impacts of depressed LV function on the surgical outcomes of this patient group. Second, participants in the study were not randomly enrolled, which may have led to some selection bias. PS matching was applied to adjust for differences in baseline characteristics to control potential confounders in the dataset. Although PS matching was used, confounders and selection biases between groups cannot be eliminated. Third, due to the retrospective and observational nature of the study, the dynamic monitoring of changes in left ventricular geometry over time was not feasible. Lastly, the assessments of the patient’s quality of life and major adverse cardiovascular events were not conducted during the follow-up period.

## 5. Conclusions

Compared to moderate IMR patients with EF ≥40%, the current study demonstrated that patients with EF <40% had similar midterm outcomes and surgical mortality, but experienced a higher incidence of prolonged ventilation. Depressed LV function may be not associated with surgical or midterm mortality.

## Data Availability

The datasets used and/or analyzed during the current study are available from the corresponding author on reasonable request.
